# Lithium-Ion Battery Solid Electrolytes Based on Poly(vinylidene Fluoride)–Metal Thiocyanate Ionic Liquid Blends

**DOI:** 10.1021/acsapm.2c00789

**Published:** 2022-07-13

**Authors:** João P. Serra, Arkaitz Fidalgo-Marijuan, João C. Barbosa, Daniela M. Correia, Renato Gonçalves, José M. Porro, Senentxu Lanceros-Mendez, Carlos M. Costa

**Affiliations:** † Physics Centre of Minho and Porto Universities (CF-UM-UP), University of Minho, Braga 4710-057, Portugal; ‡ Laboratory of Physics for Materials and Emergent Technologies, LapMET, University of Minho, Braga 4710-057, Portugal; § BCMaterials, Basque Center for Materials, Applications and Nanostructures, UPV/EHU Science Park, 48940 Leioa, Spain; ∥ Department of Organic and Inorganic Chemistry, University of the Basque Country (UPV/EHU), 48940 Leioa, Spain; ⊥ Centre of Chemistry, University of Trás-os-Montes e Alto Douro, 5000-801 Vila Real, Portugal; # Centre of Chemistry, University of Minho, 4710-057 Braga, Portugal; ∇ Ikerbasque, Basque Foundation for Science, 48009 Bilbao, Spain; ○ Institute of Science and Innovation for Bio-Sustainability (IB-S), University of Minho, 4710-057 Braga, Portugal

**Keywords:** magnetic ionic liquids, poly(vinylidene fluoride), blends, solid polymer electrolyte, solid-state lithium-ion batteries

## Abstract

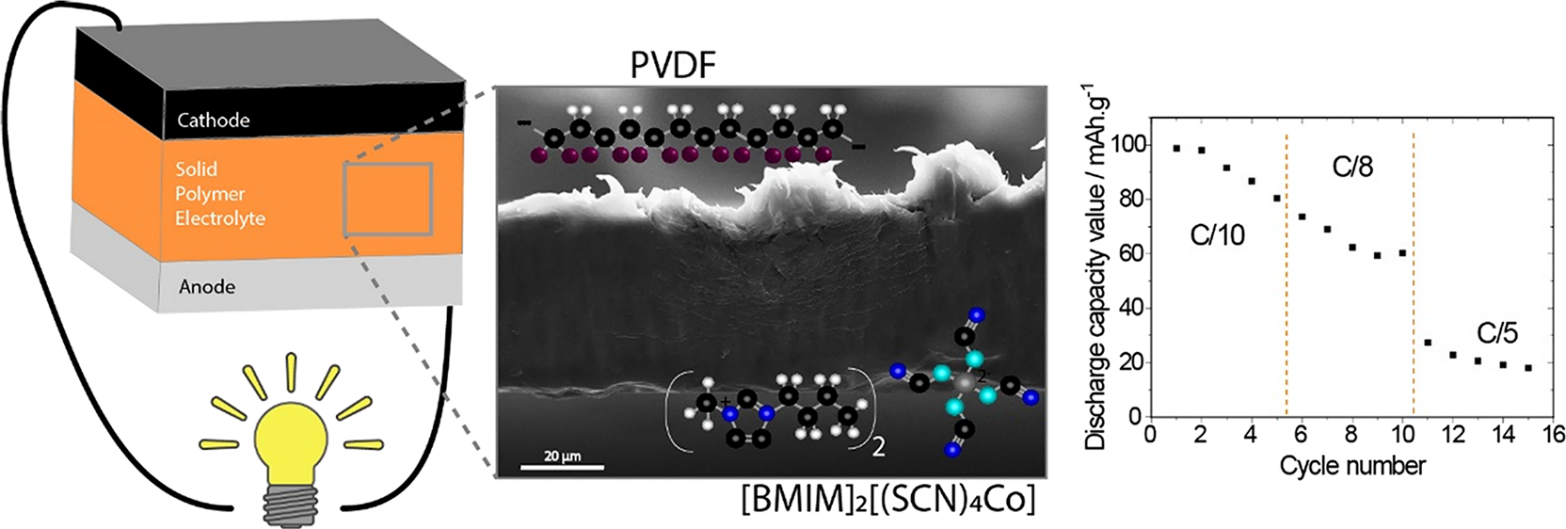

Solid polymer electrolytes (SPEs) are required to improve battery safety through the elimination of the liquid electrolyte solution in current batteries. This work is focused on the development of a hybrid SPE based on poly(vinylidene fluoride), PVDF, and 1-butyl-3-methylimidazolium cobalt(II) isothiocyanate, [BMIM]_2_[(SCN)_4_Co] magnetic ionic liquid (MIL), and its battery cycling behavior at room temperature. The addition of MIL in filler contents up to 40 wt % to the PVDF matrix does not influence the compact morphology of the samples obtained by solvent casting. The polar β-phase of PVDF increases with increasing MIL content, whereas the degree of crystallinity, thermal degradation temperature, and mechanical properties of the MIL/PVDF blends decrease with increasing MIL content. The ionic conductivity of the MIL/PVDF blends increases both with temperature and MIL content, showing the highest ionic conductivity of 7 × 10^–4^ mS cm^–1^ at room temperature for the MIL/PVDF blend with 40 wt % of MIL. The cathodic half-cells prepared with this blend as SPE show good reversibility and excellent cycling behavior at different C-rates, with a discharge capacity of 80 mAh g^–1^ at a *C*/10-rate with a Coulombic efficiency of 99%. The developed magnetic SPE, with excellent performance at room temperature, shows potential for the implementation of sustainable lithium-ion batteries, which can be further tuned by the application of an external magnetic field.

## 1 Introduction

Taking into account the constant population growth and the increasing use of resources, two of the most important issues to be tackled by modern society are related to energy and the environment.^1^ The demand for energy is increasing to satisfy the life quality of the population,^2^ and much of the world’s energy production is based on the use of fossil fuels, with their use being responsible for a large production of CO_2_ and others greenhouse gases, with the consequent influence in climate change.^3^

The energy transition to renewable energy sources represents an important contribution to address energy and environmental concerns. The investment in renewable energy reduces dependence on fossil fuels, thus enabling the production of “cleaner” energy.^4,5^ The major problem associated with the majority of renewable energy production is their intermittence and dependence on favorable environmental factors for efficient energy production.^6,7^ The absence of wind affects the production of energy from wind turbines; the low sunlight intensity affects the energy production by photovoltaic panels; and low water storage and low flow also affect energy production through hydropower, which can compromise the normal and constant supply of energy.^8^ To solve these problems, one of the possible solutions involves the coupling of energy storage systems to energy production systems, in which during the energy production and low energy consumption peaks, the system would allow energy storage for higher energy demanding peaks, avoiding also energy supply failures.^9−11^

Batteries are among the most used energy storage systems worldwide and are based on the transformation of chemical energy into electrical energy and vice versa.^12^ Among the most relevant battery types used nowadays are lithium-ion batteries (LIBs) due to their great energy and power densities and excellent electrochemical performance, compact size and low weight, low self-discharge, and long service life.^13,14^ All these characteristics make this type of batteries one of the most used today in a wide diversity of applications, ranging from mobile phones and computers to electric cars.^15^ LIBs consist of two electrodes, the cathode and the anode, which are usually separated by a membrane that is soaked in an electrolyte solution.^16^

The electrolyte solution is typically composed of lithium salts dissolved in a volatile solution with organic components and is flammable and harmful to the environment, which represents a safety problem for humans and the environment, while also presenting danger of overheating and ignition.^17,18^ One of the solutions to overcome this problem is the replacement of the separator/electrolyte system with highly conductive solid polymer electrolytes (SPEs), which are expected to integrate the next generation of batteries.^19^ SPEs comprise different categories: dry solid polymer electrolytes (dry-SPEs), single-ion conducting polymer electrolytes, and polymer-in-salt systems (rubbery electrolytes), whose current main drawbacks are a low ionic conductivity value and their interfacial interaction with the electrodes.^20^ SPEs consist of a polymeric matrix accompanied by one or more fillers. These fillers are essential to provide ionic conductivity to the matrix and to provide mechanical consistency to the SPE.^21^ The most used polymers for SPE development are poly(vinylidene fluoride) (PVDF)^22,23^ and its copolymers with hexafluoropropylene (HFP)^24^ and poly(ethylene oxide) (PEO),^25^ among others. In relation to the fillers, the most commonly used materials are lithium salts such as lithium tetrafluoroborate (LiBF_4_), lithium perchlorate (LiClO_4_), lithium hexafluorophosphate (LiPF_6_), and lithium bis(trifluoromethanesulfonyl)imide (LiTFSI),^26^ carbon-based materials (graphene oxide and carbon nanotubes), and particulate materials such as barium titanate (BaTiO_3_), titanium dioxide (TiO_2_),^27^ and ionic liquids (ILs),^28^ among others. The SPE must present a minimum ionic conductivity in the range of >10^–5^–10^–4^ S cm^–1^ to be used in LIBs.

In particular, ILs are eco-friendly materials with interesting properties for SPE applications, such as high ionic conductivity, low vapor pressure, and consequently, non-volatility and high thermal and chemical stability.^29^ Recently, SPEs based on ILs/PVDF and its copolymers have been developed with different ILs: 1-ethyl-3-methylimidazolium bis(trifluoromethylsulfonyl)imide ([EMIM][TFSI]) and 1-butyl-3-methylimidazolium thiocyanate ([BMIM][SCN]). The [BMIM][SCN]/PVDF-HFP SPE with 40 wt % IL content presents an ionic conductivity of 0.15 mS cm^–1^ and a discharge capacity of 124 mAh g^–1^ at a *C*/8-rate.^28^ A specific class of ILs is magnetic ionic liquids (MILs), which include paramagnetic compounds (transition metals like cobalt (Co), iron (Fe), or manganese (Mn)) in their structure (cations or anions). The present work proposes the development of blends based on PVDF with MILs for SPE application due to the fact that the magnetic field allows to minimize battery aging, improves the ionic transport through the magnetohydrodynamic force, and reduces the growth of the solid electrolyte interface (SEI).^30^ The blends were prepared by the solvent casting technique with varying MIL content. The selected MIL was the 1-butyl-3-methylimidazolium cobalt(II) isothiocyanate, [BMIM]_2_[(SCN)_4_Co]. The morphology, physical–chemical, mechanical, magnetic, and electrochemical properties of the MIL/PVDF blend films were evaluated, and cathodic C-LiFePO_4_ half-cells with the blend films were fabricated to obtain the charge–discharge characteristics of these batteries at room temperature.

## 2 Experimental Details

### 2.1 Materials

Poly(vinylidene fluoride) (PVDF, Solef 6010, Mw = 352–600 kDa and Solef 5130, Mw = 1000–1300 kDa), carbon black (Super P-C45), and C-LiFePO_4_ (LFP) were supplied from Solvay, Timcal Graphite & Carbon, and Phostech Lithium, respectively. The solvents *N*-methyl-2-pyrrolidone (99%) (NMP) and *N*,*N*-dimethylformamide (99%) (DMF) were purchased from Merck and the magnetic ionic liquid (MIL, 1-butyl-3-methylimidazolium cobalt(II) isothiocyanate, [BMIM]_2_[(SCN)_4_Co], from Iolitec.

### 2.2 Film Preparation

The polymer blends were produced by a solvent casting process, following the experimental methodology described in ref (31). Different amounts of MIL (0, 10, 20, and 40 wt %) were placed under magnetic stirring at 40 °C in a DMF solution. Subsequently, PVDF was added to the solution in a polymer to DMF ratio of 15/85 wt % and kept under mechanical stirring until complete dissolution of the polymer was achieved (120 min). Finally, the solution was placed on a glass substrate by a doctor blade (gap size of 300 μm) and the solvent was evaporated in an oven (P-Selecta) at 210 °C for 10 min. Under these specific conditions, the crystallization of neat PVDF typically occurs in the non-polar α-phase.^31^

**Figure 1:**
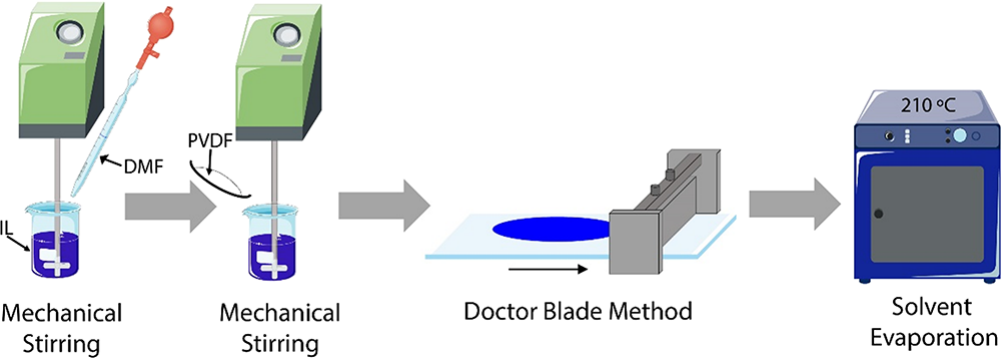
Schematic representation of the preparation methodology of the MIL/PVDF blends.

Figure 1 displays a schematic representation of the used methodology for the preparation of the MIL/PVDF blends.

### 2.3 Samples Characterization

Surface morphology of the MIL/PVDF blends and elemental analysis were obtained by scanning electron microscopy (SEM) with a Carl Zeiss EVO-40 special edition setup equipped with a secondary electron and backscattered electron detector and with an EDS elemental analyzer. The accelerating voltage of the measurements was 20 kV. A coating of a thin gold layer was applied to the samples before surface studies, in a sputter coater (SC502), Polaron, for 120 s under <10^–4^ bar pressure and 10 mA current.

The polymer phase was determined by Fourier transform infrared spectroscopy (FTIR) using a Jasco FT/IR-6100 setup in the attenuated total reflection (ATR) mode. Measurements with a resolution of 4 cm^–1^ and after 64 scans were performed from 4000 to 600 cm^–1^.

The quantification of the samples’ α*-* and β*-*phase content was based on the characteristic bands at 766 and 840 cm^–1^, corresponding to the α- and β-phases of the polymer^32^ and the application of eq 1:^32^
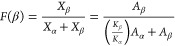
1in which *F*(β) is the β*-*phase content, *A*_α_ and *A*_β_ represent the absorbances at 766 and 840 cm^–1^, corresponding to the α*-* and β*-*phases of PVDF, and *K*_α_ and *K*_β_ are the reported absorption coefficients at the aforementioned wave numbers with *X*_α_ and *X*_β_ representing the degree of crystallinity of each phase. The value of *K*_α_ is 6*.*1 × 10^4^ cm^–2^ mol^–1^, and that of *K*_β_ is 7*.*7 × 10^4^ cm^–2^ mol^–1^.^33^

Differential scanning calorimetry (DSC) measurements were performed in a Perkin-Elmer DSC 6000 instrument using a flowing nitrogen atmosphere in a temperature range between 30 and 200 °C at a heating rate of 10 °C min^–1^. The testing was made using 40 μL aluminum pans with perforated lids that allowed the release of decomposition products during the testing.

The crystallinity degree of the MIL/PVDF blends was obtained from the DSC scans after eq 2:
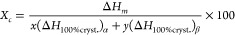
2where *x* is the weight fraction of the α-phase, *y* is the weight fraction of the β-phase (calculated from the FTIR results), and (Δ*H*_100%crystalline_)_α_ and (ΔH_100%crystalline_)_β_ are the characteristic melting enthalpies of pure crystalline α-PVDF and β-PVDF, which are 93.04 and 103.4 J g^–1^, respectively, as reported in the literature.^34^

Thermogravimetric (TGA) analysis was carried out in a TA/SDTA 851e Mettler Toledo apparatus between 25 and 800 °C at 10 °C min^–1^ and under a constant air flow of 50 mL min^–1^.

The stress–strain mechanical characteristic response of the samples was obtained with a TST350 tensile testing setup (Linkam Scientific Instruments) at room temperature and at a strain rate of 15 mm s^–1^.

The magnetic hysteresis loops of the MIL/PVDF blends were evaluated with a Micro-Sense EZ7 VSM (vibrating sample magnetometer) by sweeping the magnetic field between −4 and 4 kOe.

Impedance spectroscopy measurements were taken at open-circuit voltage in an Autolab PGSTAT-12 (Eco Chemie) equipment between 20 and 80 °C in the frequency range between 500 mHz and 65 kHz, using a constant volume support. The sample was placed between gold blocking electrodes located within a Büchi TO 50 oven. The ionic conductivity (σ_i_) of the samples was calculated by eq 3:
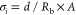
3where *R*_b_ is the bulk resistance of the sample, *d* is its thickness, and *A* is its area.

The temperature (*T*) dependence of the ionic conductivity follows the Arrhenius equation in the measured range:
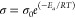
4

in which *E_a_* represents the apparent activation energy, *R* is the gas constant (8.314 J mol^–1^ K^–1^), and σ_0_ is a pre-exponential factor.

The electrochemical stability of the MIL/PVDF blends was tested in a two-electrode cell configuration, using a gold microelectrode as the working electrode and a lithium disk (Aldrich, 99.9%; 9 mm diameter, 0.75 mm thick) as the counter electrode. Cyclic voltammetry tests were performed within a dry argon-filled glove-box using an Autolab PGSTAT-12 (Eco Chemie) apparatus at a scan rate of 100 mVs^–1^.

### 2.4 Cathode Electrode and Battery Preparation. Battery Cycling Evaluation

The cathode was prepared using an 80:10:10 ratio, in which 80 wt % is for C-LiFePO_4_, 10 wt % for carbon black and 10 wt % for PVDF 5130 in 2.25 mL of NMP for 1 g of solid material. The detailed description of the electrode preparation procedure is reported in ref (35). The prepared slurry was casted on an aluminum foil using a doctor-blade, and the electrode was dried at 80 °C for 2 h. The obtained active mass loading was approximately 1.2 mg cm^–2^.

Li/C-LiFePO_4_ half-cells were assembled using Swagelok cells in a home-made glove box under an argon atmosphere, and the MIL/PVDF blends were used as SPE (10 mm diameter). Lithium metal was applied as anode and the C-LiFePO_4_-based electrode as the cathode, both with a diameter of 8 mm. Charge–discharge tests at room temperature were performed in the voltage range of 2.5 to 4.2 V at current rates from *C*/10 to *C*/5 (*C* = 170 mAh g^–1^) using a Landt CT2001A Instrument.

The electrical properties of the assembled half-cells were evaluated using electrochemical impedance spectroscopy (EIS), before and after cycling, with an Autolab PGSTAT12 equipment, at the frequency range from 10 mHz to 1 MHz with an AC voltage amplitude of 10 mV.

## 3 Results and Discussion

### 3.1 Morphological and Physicochemical Characterization

#### 3.1.1 MIL/PVDF Blends Morphology and Polymer Phase

**Figure 2:**
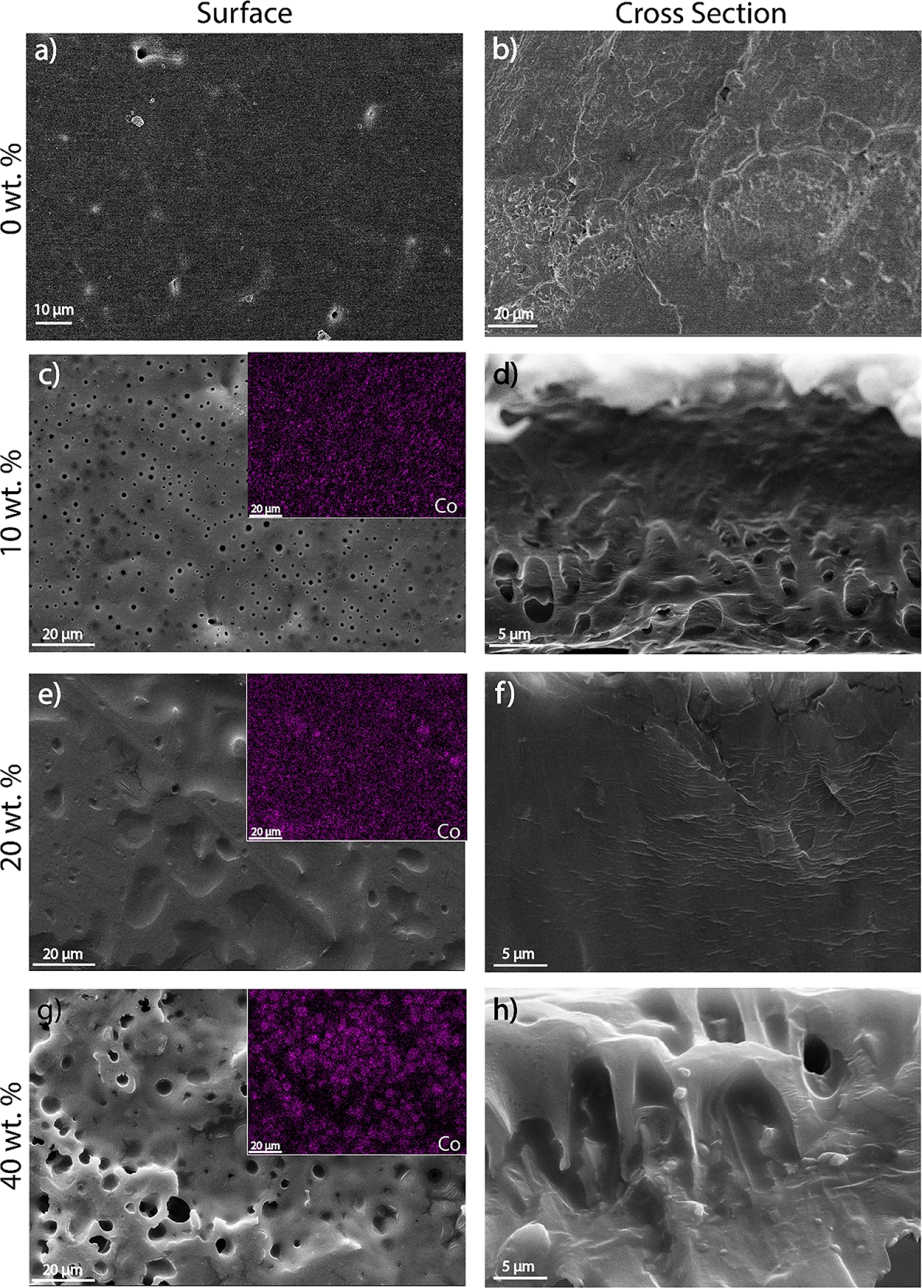
SEM images (surface and cross section) of the MIL/PVDF blends with distinct IL contents (a, b) 0, (c, d) 10, (e, f) 20, and (g, h) 40 wt %. Insets: EDS images for the cobalt element in the corresponding MIL/PVDF blends.

The effect of MIL content on the PVDF blends morphology is shown in Figure 2 through representative surface and cross-section SEM images. Neat PVDF (Figure 2a,b) shows the typical compact morphology of PVDF crystallized after solvent evaporation above the melting temperature. Solvent evaporation at *T* = 210 °C improves polymer chain diffusion, which occupies the free space previously filled by the solvent.^36^

The incorporation of distinct MIL contents (10, 20, and 40 wt %) into the PVDF matrix, induces changes in the samples’ surface with respect to the pristine polymer as can be observed in Figure 2hc–. MIL/PVDF blends with 10 and 20 wt % filler content present a small surface roughness with a well-defined spherulitic structure, while for the blend with 40 wt %, the presence of small pores on its surface is evidenced (Figure 2g). The obtained surface microstructure is ascribed to the electrostatic interaction that occurs between the IL and the polar DMF solvent, leading to the migration of some IL to the sample’s surface throughout the solvent evaporation process.

The cross section of the samples shows that, independently of the filler content, all samples present a compact morphology (Figure 2d–h), such as a neat PVDF polymer.

Further, energy dispersive spectroscopy (EDS) measurements show the uniform distribution of cobalt (purple color), a constituent element of the MILs, independently of the filler content (insets of Figure 2c,e,g).

Increasing filler content leads to the formation of small MIL agglomerates, being higher for the MIL/PVDF blends with a 40 wt % filler content. These agglomerates are nevertheless well distributed across the sample, as demonstrated by the EDS images shown in Figure 2g.

**Figure 3:**
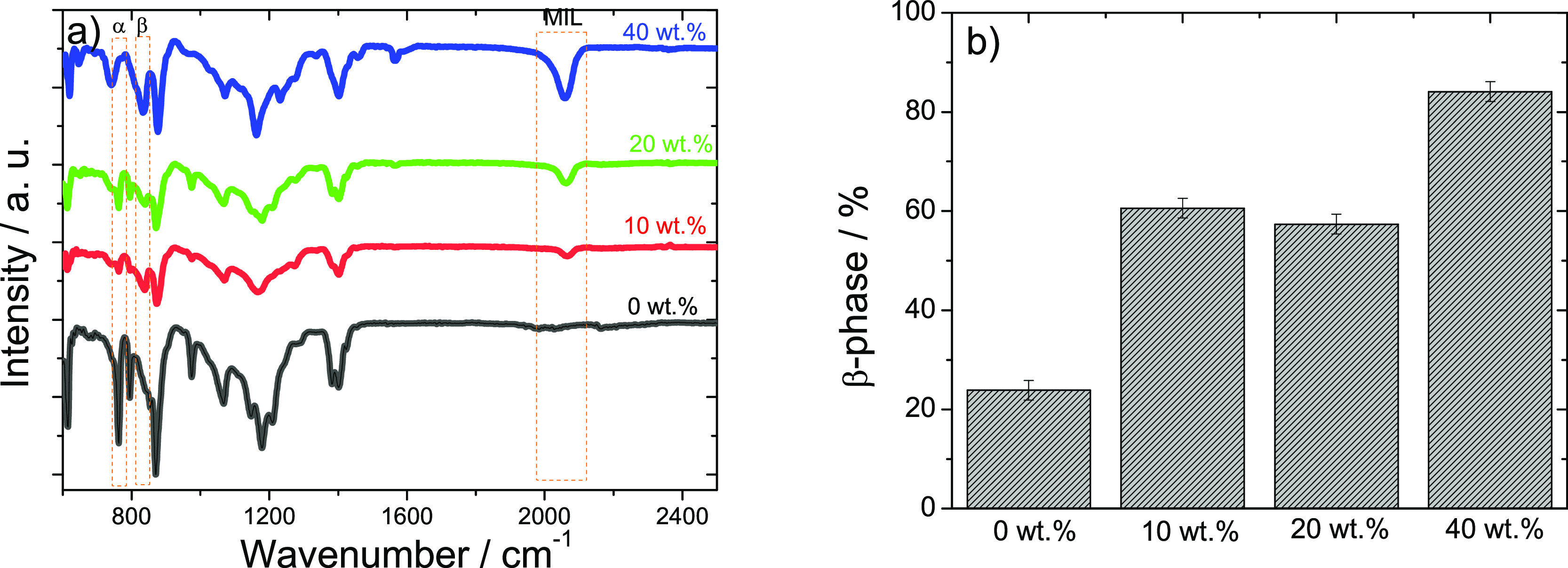
(a) FTIR spectra and (b) β-phase content of the PVDF blend samples comprising different MIL contents.

The determination (Figure 3a) and quantification (Figure 3b) of the crystalline phase of PVDF in the different samples were assessed using FTIR-ATR measurements.

The main characteristic PVDF bands of the α- and β-phases (766 and 840 cm^–1^, respectively) are shown in Figure 3a. Independently of the amount of MIL present in the PVDF polymer, the polymer crystallizes in a mixture of both crystalline phases,^32^ but the band corresponding to the β-phase (840 cm^–1^) increases with increasing MIL content. Other absorption bands characteristic of the α-phase (796, 855, and 976 cm^–1^) and β-phase (1232 cm^–1^) are also identified in the spectra of Figure 3a. Furthermore, a vibration band at 2050 cm^–1^ that corresponds to the thiocyanate anion related to the C–N stretching^37^ is also detected in the blends, which increases in intensity with increasing MIL loading within the polymer host.

The polar β-phase content (%) of the different samples was calculated using eq 1, with the results shown in Figure 3b. It is observed that the inclusion of MIL into the polymer matrix leads to a strong increase of the polymer crystallized fraction in the polar β-phase, leading to electroactive phase contents above 80% for the 40 wt % MIL content sample. The preferential crystallization of the polymer in the β-phase in the presence of the MIL is attributed to the ion–dipole interactions, leading the PVDF chains to crystallize in a preferential *all-trans* conformation.^38^

#### 3.1.2 Thermal, Mechanical, and Magnetic Analysis

**Figure 4:**
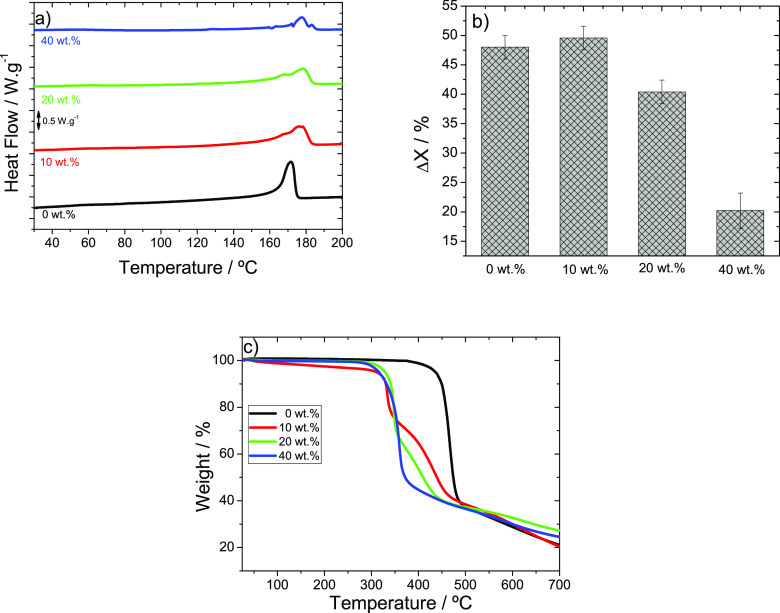
DSC thermograms of PVDF and MIL/PVDF blends incorporating different IL contents (10, 20, and 40 wt %) from (a) 30 to 200 °C, (b) crystallinity degree, and (c) TGA thermograms of the different samples.

The effect of MIL content in the thermal properties of the samples was studied by DSC and TGA thermograms (Figure 4).

Figure 4a presents the DSC thermograms of the samples from 30 to 200 °C, in order to address the effect of the presence of the IL in the melting of PVDF. Neat PVDF is characterized by a melting peak at 171 °C.^32^ The inclusion of the MIL in the MIL/PVDF blends leads to a double endothermic peak that becomes more evident with increasing IL content, which indicates the existence of the two crystal structures (α- and β-phase), as previously observed in the FTIR-ATR spectra (Figure 3).^39^ The endothermic peak at 177 °C shows that the PVDF melting temperature is shifted to higher temperatures with increasing MIL content, which is ascribed to the larger β-phase content.^32^ The crystallinity degree (Δ*X*) of the MIL/PVDF blends was calculated using eq 2, and the results are shown in Figure 4b, showing that Δ*X* decreases with the incorporation and the increase of the MIL content except for the 10 wt % filler content sample, in which MIL acts a nucleation agent. These facts are indicative of the strong interactions between the MIL with the PVDF chains that hinder polymer crystallization for the larger filler contents, whereas for smaller filler contents, they act as nucleation zones for polymer crystallization.^38,40^

Figure 4c shows the TGA thermograms of the MIL/PVDF blends. Neat PVDF shows a single maximum degradation step at nearly 450 °C, which corresponds to the degradation of the carbon–hydrogen (C–H) and carbon–fluorine (C–F) bonds.^41^ For MIL/PVDF blends, two degradation steps are detected at ∼300 and 400 °C, respectively, except for the blend with 40 wt % MIL content, attributed to the degradation temperature of the MIL^37^ and the PVDF polymer,^42^ respectively. It is interesting to observe that the thermal degradation of PVDF in the MIL/PVDF blends has a strong shift to lower temperatures, slightly dependent on the MIL content, being the strong ion–dipole interactions between MIL and polymer responsible to this effect.^43^

**Figure 5:**
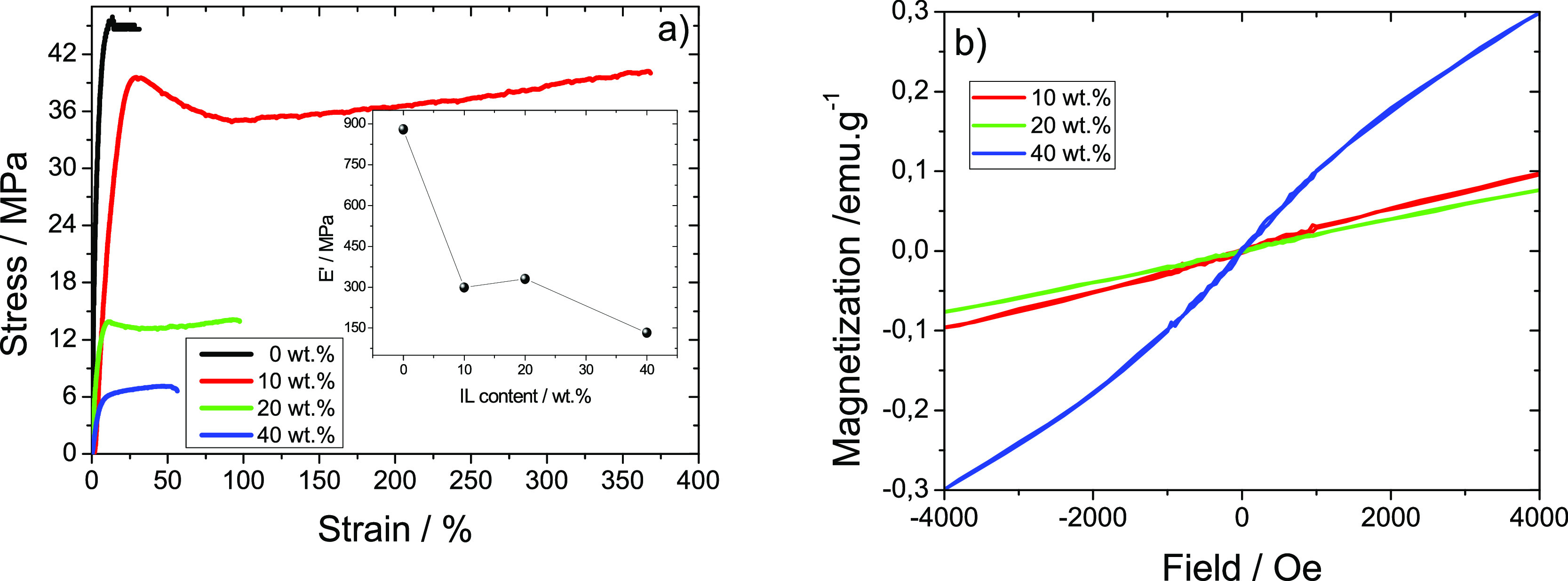
(a) Stress–strain plots (inset, Young modulus value as a function of MIL content in the sample) and (b) room-temperature magnetic hysteresis loops for PVDF and MIL/PVDF blends with 10, 20, and 40 wt % of MIL.

Figure 5a shows the stress–strain analysis of neat PVDF and MIL/PVDF blends. All samples display the characteristic thermoplastic mechanical behavior of PVDF, with well-defined elastic, yielding, and plastic regions.^44^ The Young modulus was determined by the tangent method at 3% of elongation within the elastic region, obtaining maximum and minimum *E*′ values of 880 and 133 MPa for PVDF and the MIL/PVDF blends with 40 wt %, respectively, according to the inset in the Figure 5a. The obtained *E*′ values indicate that the inclusion of the MIL into the PVDF matrix leads to a plasticizing behavior within the blend sample.

Furthermore, it is shown that the Young modulus and yield stress are reduced with increasing MIL content in the PVDF polymer, which is attributed to the decrease of the degree of crystallinity and the plasticizing effect of the MIL. Moreover, it is noted that MIL addition improves the elongation at break of the samples, compared to neat PVDF. This behavior depends on the MIL content and is higher when 10 wt % of MIL is added to the PVDF with a maximum strain percentage around 375%.

The magnetic properties of the MIL/PVDF blends were evaluated by vibrating sample magnetometry measurements at room temperature. Figure 5b shows the magnetization as a function of the magnetic field applied to each of the blends studied here. While magnetization follows a linear paramagnetic correlation with the magnetic field for the lower content (10 and 20 wt % MIL), the 40 wt % MIL blend starts to show an incipient ferromagnetic behavior, while the magnetization of the blends increases with the wt % MIL. This is a consequence of the higher Co content on the higher wt % MIL blend.

### 3.2 Ionic Conductivity and Electrochemical Stability Window

The electrochemical performance of the MIL/PVDF blends was assessed by impedance spectroscopy.

**Figure 6:**
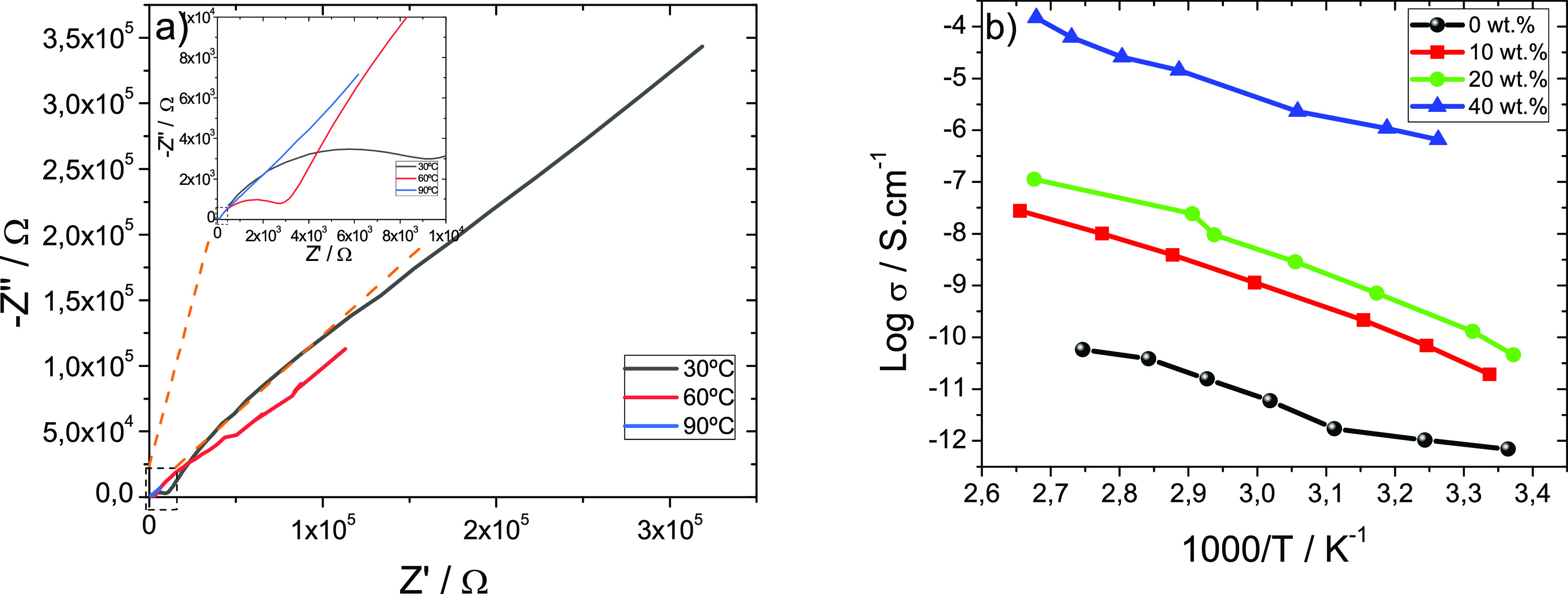
(a) Nyquist plots of the 40 wt % MIL/PVDF blends at different temperatures (30, 60, and 90 °C) and (b) temperature dependence of the ionic conductivity for the MIL/PVDF blends with different MIL contents.

Figure 6a displays the Nyquist plots of the 40 wt % MIL/PVDF blends at three different temperatures (30, 60, and 90 °C), which present two well defined regions: a semicircle at high frequency values resembling the charge transfer process and a straight line present at lower frequencies that describes the charge diffusion process.^45^

The impedance values are temperature dependent, and the semicircle related to the charge transfer process diminishes with increasing temperature. This is related to the fact that the temperature increase leads, on one hand, to faster internal modes in the polymer chains, with the bond rotations supporting intra-chain ion movements.^46^ On the other hand, increasing temperature leads to improved thermally activated dynamics of the ions of the MIL. This behavior is also representative of the ones of the MIL/PVDF blends with 10 and 20 wt % MIL contents.

The ionic conductivity was calculated from the Nyquist plot shown in Figure 6a, using eq 3, where *R*_b_ is obtained from the interception between the imaginary Z″ and real Z″ components of the impedance. Figure 6b shows the ionic conductivity variation as a function of temperature for the different MIL/PVDF blends. The ionic conductivity value (σ_i_) and the apparent activation energy (*E_a_*) were calculated by fittings to the Arrhenius equation,^47^ and the results for all samples are shown in Table 1. The room temperature ionic conductivity value for neat PVDF is 7 × 10^–10^ mS cm^–1^.

**Table 1:** Ionic Conductivity (σ_i_) and Activation Energy (*E_a_*) Values for the MIL/PVDF Blends

IL/PVDF	% wt IL	σ_i_/mS cm^–1^ (25 °C) (±3%)	*E_a_*/kJ mol^–1^ (±3%)
[BMIM]_2_[(SCN)_4_Co]	0 wt %	7 × 10^–10^	181
	10 wt %	1.9 × 10^–8^	38
	20 wt %	4.6 × 10^–8^	41
	40 wt %	7 × 10^–4^	32

Figure 6b shows that, independently of the temperature, the ionic conductivity is improved with the increase of the MIL content within the PVDF matrix due to the increase of number of charge carriers, anions, and cations of the MIL inside the polymer matrix. The highest ionic conductivity value at room temperature is obtained for 40 wt % MIL/PVDF blends with a value of 7 × 10^–4^ mS cm^–1^. In addition, Table 2 displays the ionic conductivity for the 40 wt % MIL/PVDF blend compared to the current literature on SPEs based on ILs. The present MIL shows an ionic conductivity up to 2 orders of magnitude lower than the best ones in the literature, though enough for battery applications and with the characteristic of presenting magnetic response, which can be suitable for LIBs, as it has been reported that the use of magnetic fields reduces the aging effect, inhibits SEI growth and lithium plating, and also homogenizes ionic transport through the magnetohydrodynamic force.^30^

**Table 2:** Ionic Conductivity Value for Different SPEs with ILs

polymer	components	σ_i_ (mS cm^–1^) at 25 °C	ref
PI	[Bmim][TFSI], LiTFSI	1.7 × 10^–2^	(48)
PEO, PVDF	LiTFSI, POSS-IL	80 × 10^–2^	(49)
PEO, PVDF-HFP, PC	LiTFSI, POSS-IL	39 × 10^–2^	(50)
PVDF-HFP	[BMIM][SCN]	15 × 10^–2^	(51)
PEO	LiDFOB-[EMIM][TFSI]	7 × 10^–3^	(52)
PVDF	[BMIM]_2_[(SCN)4Co]	7 × 10^–4^	this work

**Figure 7:**
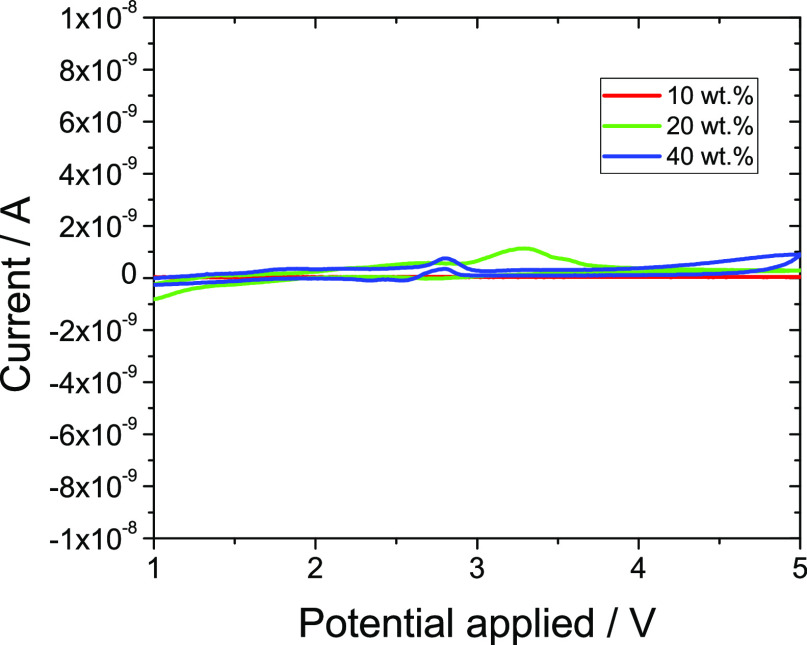
Cyclic voltammogram of the different blends at room temperature and 100 mV s^–1^.

Figure 7 shows the electrochemical stability of the MIL/PVDF blends at room temperature and at 100 mV s^–1^.

Regardless of the MIL/PVDF blends, all samples show good electrochemical stability in the 1 to 5 V range, considering the current in order of nA. Furthermore, a small anodic peak is observed in the different blends, attributed to irreversible processes within the MIL–polymer blend, which does not affect the cycling behavior considering its value in the order of nA. Finally, it is observed that the cyclic voltammgram presents a reversible behavior for the SPE, being suitable for battery applications.

### 3.3 Battery Performance

**Figure 8:**
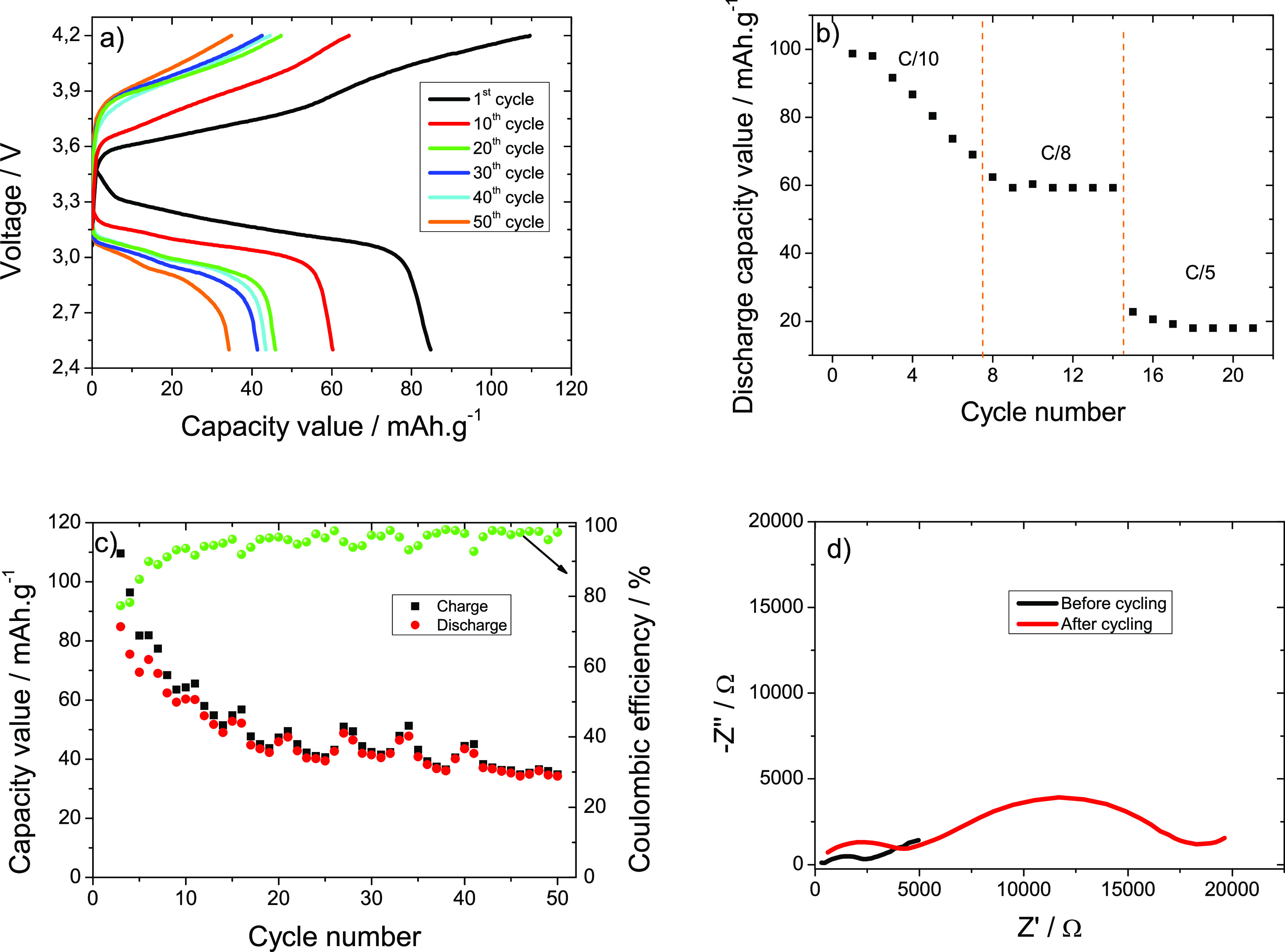
(a) First, 20th, 30th, 40th, and 50th charge and discharge room temperature cycles at a *C*/8 rate, (b) rate performance at *C*/10, *C*/8, and *C*/5 discharge rates, (c) cycling stability performance at a *C*/8 rate, and (d) impedance spectra before and after cycling for the 40 wt % MIL/PVDF SPE-based cathodic half-cells.

Taking into account that the best ionic conductivity values were achieved for the sample with 40 wt % MIL, the cycling performance was evaluated for this sample in cathodic half-cells with C-LFP. Cycling tests were obtained at room temperature at *C*/10, *C*/8, and *C*/5-rates, in the potential range between 2.5 and 4.2 V that corresponds to the voltage range of the LFP structure without causing any damage to it. Figure 8a presents the typical charge–discharge profile for the 1st, 20th, 30th, 40th, and 50th cycles at a *C*/8-rate. This profile is typical of the active material C-LiFePO_4_ cathode,^53^ and the charge and discharge values decrease after the 50th cycle due to the development of the solid electrolyte interface (SEI) layer during cycling,^54^ demonstrating nevertheless a good reversibility process.

Figure 8b displays the rate performance at three different discharge rates for 7 cycles at each rate and room temperature. For each rate in the 7th cycle, the discharge capacity values are 80, 61, and 17 mAh g^–1^ at the *C* rates of *C*/10, *C*/8, and *C*/5, respectively. For the *C*/8-rate, it is concluded that the discharge capacity value continuously decreases during cycling due to the SEI formation. Furthermore, for other *C*-rates, the discharge capacity value is almost constant. Also, it is detected that the increase in the *C*-rate leads to a reduction in the discharge capacity value due to the polarization effect and the interfacial reaction resistance within the electrode.^55^

Figure 8c presents the charge/discharge capacity values at the *C*/8-rate for 50 cycles. The discharge capacity values are 72 and 30 mAh. ^–1^, respectively, in the 1st and 50th cycle, and the Coulombic efficiency is close to 99% at all cycles. It is observed that the electrochemical behavior decreases up to 15 cycles due to the SEI layer formation. It is to notice that the observed charge/discharge capacity value wave-like fluctuations are not dependent on the SPE but on the daily temperature fluctuations of the laboratory. Further, excellent Coulombic efficiency is observed after the 50 cycles, which indicates good reversibility in the process.

Figure 8d shows the EIS spectra for the batteries before and after the cycling process.

For both spectra, the Nyquist plot is composed by two characteristic semicircles at high and medium frequencies that represent the contact film resistance and the resistance from the charge-transfer reaction resistance and a straight line at the low frequency region that represents the Li^+^ diffusion process characterized by the Warburg element.^56^

It is observed that the total resistance (ohmic resistance, contact film resistance, and charge-transfer reaction resistance) varies before and after cycling, with values of 2450 and 18,285 Ω, respectively. This increase is due to the formation of a SEI layer in the cycling process.^54^

It is to notice that most current literature studies on polymer matrix SPE show battery performance at temperatures above 50 °C,^57−59^ which limits its applicability, whereas in the present case, the battery performance is evaluated and presented at room temperature.

In summary, considering the battery performance at room temperature for this SPE with IL, this work demonstrates a step forward in the development of SPEs for application in lithium-ion batteries. The use of MILs in LIBs is a promising approach due to its possibility to reduce the aging effects and achieve higher battery performance resulting from lower SEI growth and ion transport homogenization, when a magnetic field is applied.^30^

## 4 Conclusions

Solid polymer electrolytes, SPEs, based on PVDF and the [BMIM]_2_[(SCN)_4_Co] magnetic ionic liquid, MIL, have been obtained by the solvent casting technique at 210 °C, and the influence of MIL content on morphology, physical, thermal, mechanical, magnetic, and electrochemical properties has been evaluated.

MIL/PVDF blends show a compact morphology independently of the filler content and the β-phase content. Their thermal and mechanical properties are influenced by the MIL content in the PVDF matrix. The β-phase content is dependent on the MIL content due to the electrostatic interactions between the MIL and the PVDF polymer chains. The degree of crystallinity of the blends decreases from 48 to 20% with increasing MIL content. Similarly, higher MIL incorporation rates lead to a reduced thermal stability of the samples. Independently of the MIL content, its incorporation into the PVDF matrix leads to a plasticizing effect, reducing the Young modulus from 880 to 133 MPa. In addition, a crossover from a paramagnetic to an incipient ferromagnetic behavior in the blends is observed as the MIL content is increased.

The ionic conductivity of the samples depends on the MIL content and the temperature. The highest room temperature ionic conductivity (7 × 10^–4^ mS cm^–1^) is achieved for the MIL/PVDF blends with 40 wt %. Also, these blends show excellent electrochemical stability in the potential window up to 5 V.

Battery performance for the MIL/PVDF blends with a 40 wt % filler content in cathodic half-cells at room temperature shows good reversibility, and the discharge capacity values are 80, 61, and 17 mAh g^–1^ at the *C* rates of *C*/10, *C*/8, and *C*/5, respectively, demonstrating excellent cycling behavior at room temperature and 99% of Coulombic efficiency. Furthermore, after 50 cycles at the *C*/8-rate, the cycle behavior is nearly constant, demonstrating the suitability of this SPE for the next generation of LIBs able to take advantage of the application of magnetic fields to improve battery performance by reducing aging and SEI growth as well as to improve ion transport through the magnetohydrodynamic force.
